# Skeletal muscle feature of different populations in large yellow croaker (*Larimichthys crocea*): from an epigenetic point of view

**DOI:** 10.3389/fmolb.2024.1403861

**Published:** 2024-07-02

**Authors:** Shangwei Xie, Lihua Jiang, Weihua Song, Jialang Zheng, Yifan Liu, Shun Chen, Xiaojun Yan

**Affiliations:** ^1^ National Engineering Research Center of Marine Facilities Aquaculture, College of Fisheries, Zhejiang Ocean University, Zhoushan, Zhejiang Province, China; ^2^ Nanji Archipelago National Marine Nature Reserve Administration, Wenzhou, Zhejiang Province, China

**Keywords:** *Larimichthys crocea*, DNA methylation, skeletal muscle, epigenetics, environmental adaptation

## Abstract

Fish skeletal muscle is composed of well-defined fiber types. In order to identify potential candidate genes affecting muscle growth and development under epigenetic regulation. Bisulfite sequencing was utilized to analyze and compare the muscle DNA methylation profiles of *Larimichthys crocea* inhabiting different environments. The results revealed that DNA methylation in *L. crocea* was predominantly CG methylation, with 2,396 differentially methylated regions (DMRs) identified through comparisons among different populations. The largest difference in methylation was observed between the ZhouShan and JinMen wild populations, suggesting that *L. crocea* may have undergone selection and domestication. Additionally, GO and KEGG enrichment analysis of differentially methylated genes (DMGs) revealed 626 enriched GO functional categories, including various muscle-related genes such as *myh10, myf5, myf6, ndufv1, klhl31, map3k4, syn2b, sostdc1a, bag4, and hsp90ab*. However, significant enrichment in KEGG pathways was observed only in the JinMen and XiangShan populations of *L. crocea*. Therefore, this study provides a theoretical foundation for a better understanding of the epigenetic regulation of skeletal muscle growth and development in *L. crocea* under different environmental conditions.

## 1 Introduction

Local adaptation represents one of the primary mechanisms facilitating organismal adjustments to environmental shifts or novel habitats (Zhao et al., 2018). Within a given species, organisms evolve and manifest diverse phenotypes in response to local environmental cues, thereby achieving local adaptation. Studies propose that environmentally induced epigenetic mutations may be linked to sensory and response systems, thereby influencing phenotypic plasticity. DNA methylation variations may arise in response to environmental changes affecting methylation processes (Zhang et al., 2019), thereby contributing to local adaptation in animals. This adaptive strategy may represent the most advantageous choice for species grappling with environmental fluctuations. Research on sand cicada (*Magicicada spp*) populations from five coastal locations in South Africa indicates a negative correlation between genome-dependent epigenetic mutations and genetic diversity. However, populations in transitional areas experiencing high levels of environmental change may adapt to new conditions through environmentally induced epigenetic mutations. The reciprocal transplant experiments on African clawed frogs (*Xenopus laevis*), illustrating that precipitation levels in different climatic regions influence larval survival rates (Kruger et al., 2022). This study also confirmed that phenotypic plasticity and environmental adaptation contribute to phenotypic variation in tadpoles of the two populations under contrasting rainfall conditions, elucidating that population-level phenotypic changes are often associated with climatic variables. Epigenetic variation, particularly DNA methylation, entails genetic changes in gene expression and function in response to environmental or endogenous signals, resulting in physiological phenotypes manifested at higher levels of biological tissues, which cannot be explained by changes in DNA sequence. The ambient temperature exerts a pervasive influence on the biochemical, physiological functions, and behavioral patterns of ectothermic organisms.

Fish inhabit aquatic environments and are highly susceptible to changes in heterogeneous surroundings, rendering them ideal subjects for investigating the potential impact of environmental-gene interactions on the sustainable development of species. Currently, our understanding of the mechanisms and patterns of DNA methylation in fish stems not only from studies in model species like zebrafish and stickleback (Li and Liu, 2021; Liang et al., 2023), but also from research on commercially significant fish species such as rainbow trout, *Atlantic salmon*, Chinese perch, and tilapia. These studies have demonstrated that DNA methylation is often associated with the transcription of genes and transposons, and can influence gene expression regulation, thereby facilitating phenotypic variation to adapt to environmental heterogeneity. Research has indicated that temperature significantly influences the growth, development, swimming performance, and enzyme metabolism in the skeletal muscle of *Chelydra serpentina*, *Rana sylvatica*, and *Danio rerio*. In most bony fish, muscle growth and differentiation persist into adulthood, with their muscle phenotype being regulated by temperature during embryonic development. Consequently, gaining a deeper understanding of the molecular mechanisms of methylation in marine organisms is essential for comprehending their growth, development, and evolutionary processes (Koganti et al., 2021).

The *L. crocea* is a significant commercial marine fishery resource highly prized for its tender meat, which boasts a plethora of high-quality proteins, amino acids, and mineral elements. Abundant in multiple polyunsaturated fatty acids, including DHA, it commands a high economic value and is in great demand. However, due to excessive overfishing and inadequate fisheries management, the total wild population of *L. crocea* has experienced a sharp decline, leading to the inability to establish effective fishing seasons. Since the 1980s, the advancement of breeding technology has facilitated the promotion of its aquaculture (Yang et al., 2014). Nonetheless, surveys and statistics indicate that the market price of wild *L. crocea* is significantly higher than that of cultured ones due to the tender nature of wild-caught specimens. Research indicates that the main edible part and quality value of fish muscle lie in the skeletal muscle tissue, which is closely linked to muscle hardness (Stadler et al., 2011). The quality of muscle can be influenced by various endogenous factors such as genetic background, color, and fat content, as well as by environmental and exogenous factors such as feeding methods (Greenberg and Bourc’his 2019; He et al., 2020; Luo et al., 2018). Concurrently, artificial breeding may impact disease resistance, adaptability, swimming ability, and body size, potentially leading to obesity and reduced stature (Buckingham and Rigby, 2014; Song et al., 2019). Moreover, persistent challenges include variability in seedling quality, inadequate accumulation of superior germplasm resources, and a scarcity of varieties suitable for extensive aquaculture deployment. Environmental constraints further hinder the realization of an ideal distribution pattern, where *L. crocea* could be cultured in the north while being sourced from the south (Stickland and Handel, 1986; Carrió et al., 2016; Laker and Ryall, 2016). In natural marine habitats, *L. crocea* displays notable geographical diversity in its morphological characteristics, age of sexual maturity, and lifespan. Different phenotypic traits of *L. crocea* are evident across various marine areas, including variations in body length, body color, gill structure, and vertebral bones (Brunk et al., 1996; Carrió et al., 2015). These differences may be attributed to adaptive changes and survival strategies in response to diverse environmental conditions. Consequently, investigating the adaptability of *L. crocea* in different habitats holds great significance for the preservation of germplasm resources and the future sustainable development of this species.

In this study, the epigenome-wide DNA methylation patterns of *L. crocea* were constructed using BS-seq sequencing for five different populations from diverse habitats. The objective of this research was to analyze the potential impact of DNA methylation on the adaptation to artificial domestication and environmental selection at the epigenetic level. The findings of this study will contribute to our understanding of the skeletal muscle features involved in the environmental adaptation of *L. crocea*.

## 2 Materials and methods

### 2.1 Sample collection

**FIGURE 1:**
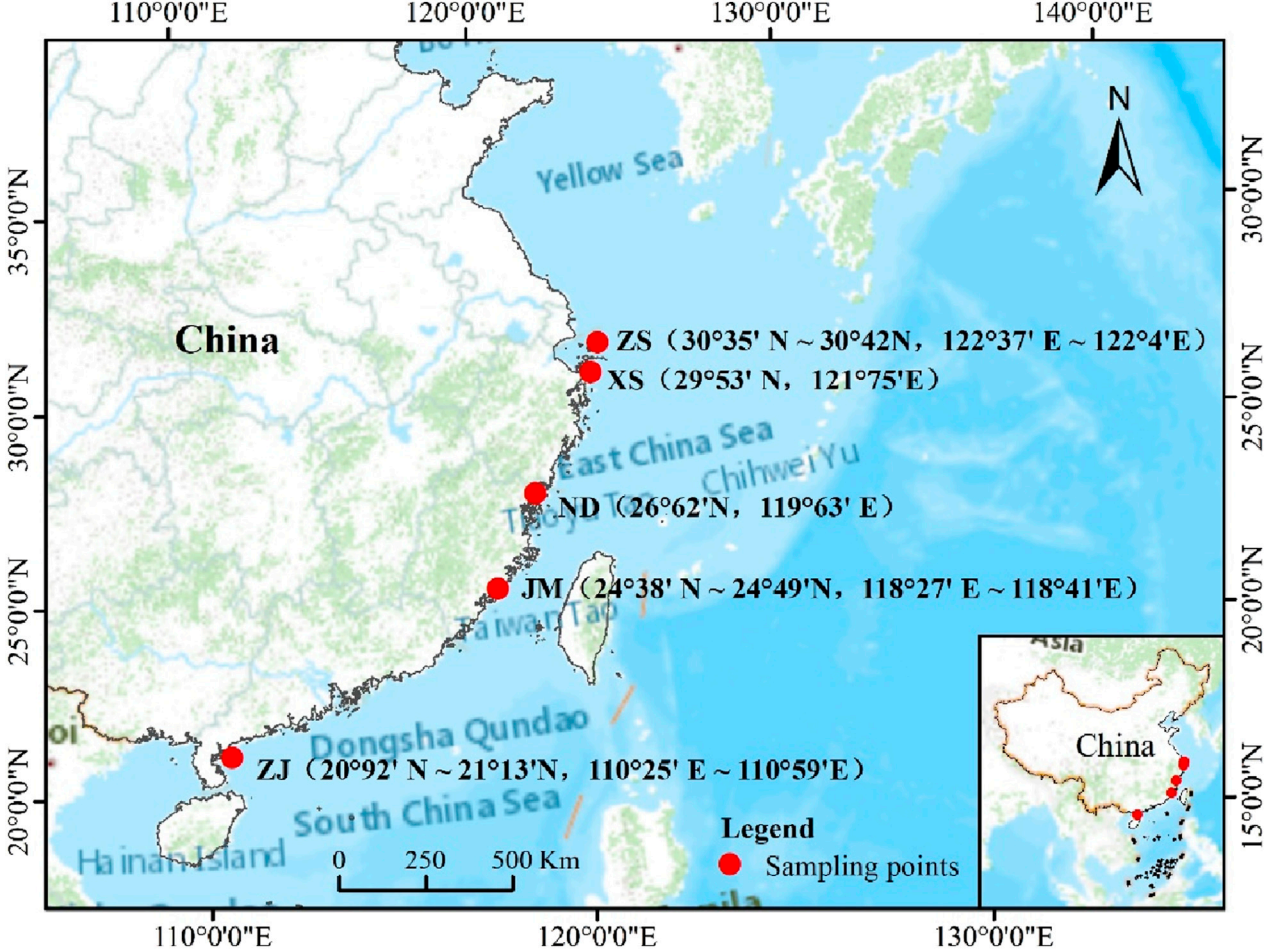
The sites of sample collection.

This experiment collected a total of five different samples of *L. crocea* from various aquaculture or wild-capture environments, including ZhouShan (30°35′N∼30°42′N, 122°37′E∼122°4′E), ZhanJiang (20°92′N∼21°13′N, 110°25′E∼110°59′E), JinMen (24°38′N∼24°49′N, 118°27′E∼118°41′E), NingDe (26°62′N, 119°63′E), and XiangShan (29°53′N, 121°75′E), sampled between 2020 and 2021. Wild-captured *L. crocea* were obtained through fishing or trawling by fishermen, while aquaculture *L. crocea* were procured from aquaculture farms (Ningde Fufa Aquatic Products Co., Ltd., Xiangshan Bay Aquatic Seedlings Co., Ltd.) The geographic locations of the sampling sites were plotted using ArcGIS software (Figure 1).

The classification of the population samples was based on the geographical divisions proposed by Liu for *L. crocea* (Liu and De Mitcheson, 2008), supplemented by expert identification of morphological characteristics. All fish used in the experiment were adult *L. crocea*, with approximately 30–45 samples collected for each population, totaling 188 samples from the five *L. crocea* populations. Muscle tissue was extracted from all samples and stored at −80°C. To minimize inter-sample variability, DNA from five to seven fish was pooled for each population sample, resulting in a total of 42 samples for whole-genome DNA methylation sequencing (Table 1).

**TABLE 1:** Summary of sampling information of *Larimichthys crocea*.

Populations	Longitude	Latitude	Individual no.	Farmed/wild
Jinmen	24°38′N∼24°49′N	118°27′E∼118°41′E	35	farmed
Zhoushan	30°35′N∼30°42′N	122°37′E∼122°4′E	35	farmed
Zhanjiang	20°92′N∼21°13′N	110°25′E∼110°59′E	30	wild
Xiangshan	29°53′N	121°75′E	36	wild
Ningde	26°62′N	119°63′E	52	wild

### 2.2 Library construction and DNA methylation sequencing

In this experiment, DNA extraction was performed using a universal column genomic DNA extraction kit, followed by ultrasonic treatment of sample DNA using Covaris S220 (BGI) to obtain DNA fragments ranging from 100 to 500 bp in length. The resulting small fragment DNA underwent 5′end phosphorylation repair, 3′end addition of “A”, and ligation to sticky end adaptors carrying “T”. Subsequently, the DNA was treated with bisulfite using the EZ DNA Methylation-GoldTM Kit, wherein unmethylated cytosines (C) were converted to uracils (U) (which become thymine (T) after PCR amplification), while methylated cytosines remained unchanged. Finally, PCR amplification was conducted to generate the final DNA library.

Following library construction, the library was quantified using Qubit 3.0 and diluted to a concentration of 1 ng/μL. The length and effective content of the library fragments were assessed using Agilent 2100, and Q-PCR was performed to ensure compliance with the specified standards. Subsequent sequencing work proceeded once the library was deemed suitable for sequencing.

### 2.3 Quality control of sequencing data and alignment of sequences

The signal set collected from Illumina HiSeq TM 4000 sequencing platform is processed by base calling to obtain the raw sequence data in FASTQ format. In order to ensure the accuracy of data analysis, the commonly used quality assessment by software FastQC (v0.11.8) (de Sena Brandine and Smith, 2019), was used for quality evaluation, and Trimmomatic (v0.36) (Bolger et al., 2014) was used to filter the raw reads to obtain clean reads for subsequent information extraction and analysis. The filtering steps are as follows: 1) removal of reads containing adapters; 2) trimming reads with quality <3 at both ends or containing N (representing an undefined base); 3) removal of low-quality reads, with a sliding window of four bases, and if the average quality value of the bases in the window is too low (<15), the reads should be trimmed from that point; 4) removal of reads with a length less than 50 nt after trimming; 5) removal of unpaired reads; 6) for low starting quantity methylation libraries, the first 10 bp are trimmed. Then, the bismark_genome_preparation program in Bismark software (v0.22.1) (Krueger and Andrews, 2011) is used to align the four parallel-converted chains with the reference genome, resulting in all alignment results. The reference genome for the L. crocea_2.0(GCA_000972845.2) used for the alignment was obtained from the Ensembl (http://asia.ensembl.org/index.html) database. Finally, the deduplicate_bismark program in Bismark is used to remove duplicate alignment results.

### 2.4 Differentially methylated regions (DMRs) identification

In this study, a comparative analysis was conducted on populations of *L. crocea* (ZS, JM, ZJ, XS, and ND) using the calmeth program in BatMeth2 to identify methylation sites throughout the genome and calculate the methylation level of individual C sites (Zhou et al., 2019). Sites with a coverage of at least four reads were marked. The methylation level of genomic elements was calculated using the metyGff program, and the MethylKit (v0.99.2) was used to perform 600 bp sliding window segmentation of the *L. crocea* genome (Akalin et al., 2012). Fisher’s exact test (Fajardo Dueñas and González Moreno, 2002) was used to compare the segmented regions (*p* < 0.01, *q* < 0.01) and identify differentially methylated regions (DMRs).

### 2.5 Principal component analysis

This study aimed to investigate the differences in DNA methylation between the *L. crocea* populations from different habitats, including wild and cultured ones. The five populations were compared to each other to identify potential differences in methylation levels. The statistical correlation between methylation levels was calculated to assess the reliability of the experiment and the adequacy of the sample selection. Methylation levels were then calculated for each bin, using a 2K bp/bin window, and subjected to principal component analysis (PCA) (Abdelhafez et al., 2020). By comparing the methylation patterns of different populations at the chromosome level, functional regions, and 2K bp upstream and downstream of genes, we could gain a better understanding of the methylation patterns and explore the relationships between them in more depth.

### 2.6 Differential methylation region annotation and gene structure analysis

This study annotated the DMRs to transcription start sites upstream 2K (upstream 2K), exons, introns, and gene end sites downstream 2K (downstream 2K) to investigate their distribution in these gene elements. The swDMR (v1.0.7) (Wang et al., 2015) software was then used to identify DMRs using a sliding window algorithm based on defined window size and step size parameters, with filtering criteria set at –window 1,000 –step Size 100 –length 100 –*p* value 0.01 –coverage 4 –fold 2 –fdr 0.05. Fisher’s exact test was used to identify genes that overlapped with DMR regions in the entire genome functional area, which were called DMR-associated genes (DMGs). Among them, genes with *q*-value < 0.05 and meth. diff ≥ 20 were identified as hypermethylated genes (hyper-DMGs), while genes with *q*-value < 0.05 and meth. diff ≤ 20 were identified as hypomethylated genes (hypo-DMGs). Genes related to skeletal muscle growth and development were selected from differentially methylated genes, and their composition elements were predicted to infer the location of the identified differentially methylated genes to determine their roles. The sequence information of *L. crocea* was obtained from the UCSC (http://www.genome.ucsc.edu/index.html) database, and the promoter and gene structure were predicted using the online website Softberry (http://www.softberry.com/berry.phtml).

### 2.7 GO and KEGG analysis

The identified differentially methylated genes were subjected to Gene Ontology (GO) and Kyoto Encyclopedia of Genes and Genomes (KEGG) enrichment analysis using the R package clusterProfiler (https://github.com/YuLab-SMU/clusterProfiler). This analysis aimed to gain a deeper understanding of the biological functions of the identified differentially methylated genes in marine bioinformatics and to more accurately explore the biological functions of gene sets, providing important reference for the development of marine bioinformatics. The reliability of the DMR enrichment to GO terms and KEGG pathways was calculated using a threshold of *p* value Cutoff = 0.05 and *q* value Cutoff = 0.05.

## 3 Results

### 3.1 Methylation sequencing information statistics

In this study, a total of 622.07 Gb of raw data was obtained from five *L. crocea* population samples. After filtering, 588.41 Gb of clean reads were obtained, accounting for 94.58% of the raw reads. Sequencing analysis revealed that both the proportion of base quality values Q20 and Q30 exceeded 90%, indicating that the sequencing quality met the requirements and provided reliable support for downstream analysis. Additionally, alignment of the reads to the *L. crocea* reference genome using Bismark software resulted in a proportion of mapped reads above 60%. Furthermore, the removal of false positive reads generated during PCR amplification was performed to improve data quality. The coverage of each single base site in the processed data was calculated, revealing that approximately 90% of bases had a depth of at least 1× in all samples, while about 80% had a depth of at least 4×. Moreover, approximately 70% of the genome exhibited a base coverage depth of at least 10×. A distribution map of the coverage depth of the whole genome was generated based on the proportion of bases in the entire genome, indicating that a large proportion of the genome had a sequencing depth of 10× or more, accounting for about 70%. These results collectively suggest that the sequencing depth and coverage of the genome were sufficient, providing reliable evidence for identifying methylation sites in future analyses.

### 3.2 Genome-wide methylation level analysis

After evaluating the methylation data, statistical analysis was conducted on the C sites, including the calculation of coverage proportions in different sequence contexts (CG, CHH, CHG, where H represents A, C, or T). Analysis of the distribution and levels of methylated C sites across the entire genome of *L. crocea* revealed that the methylation level of mCG was the highest, exceeding 88%. This was followed by mCHH, which accounted for approximately 6%–8% of methylated sites, and finally mCHG, which constituted around 2%–3% of methylated sites (Supplementary Data S3). Moreover, significant differences were observed in the methylation levels and patterns of C sites among different contexts in the entire genome. CG regions exhibited predominantly high methylation levels, whereas CHH and CHG regions displayed similar trends, characterized by low methylation and unmethylated sites.

### 3.3 Differential methylation identification and principal component analysis

**FIGURE 2:**
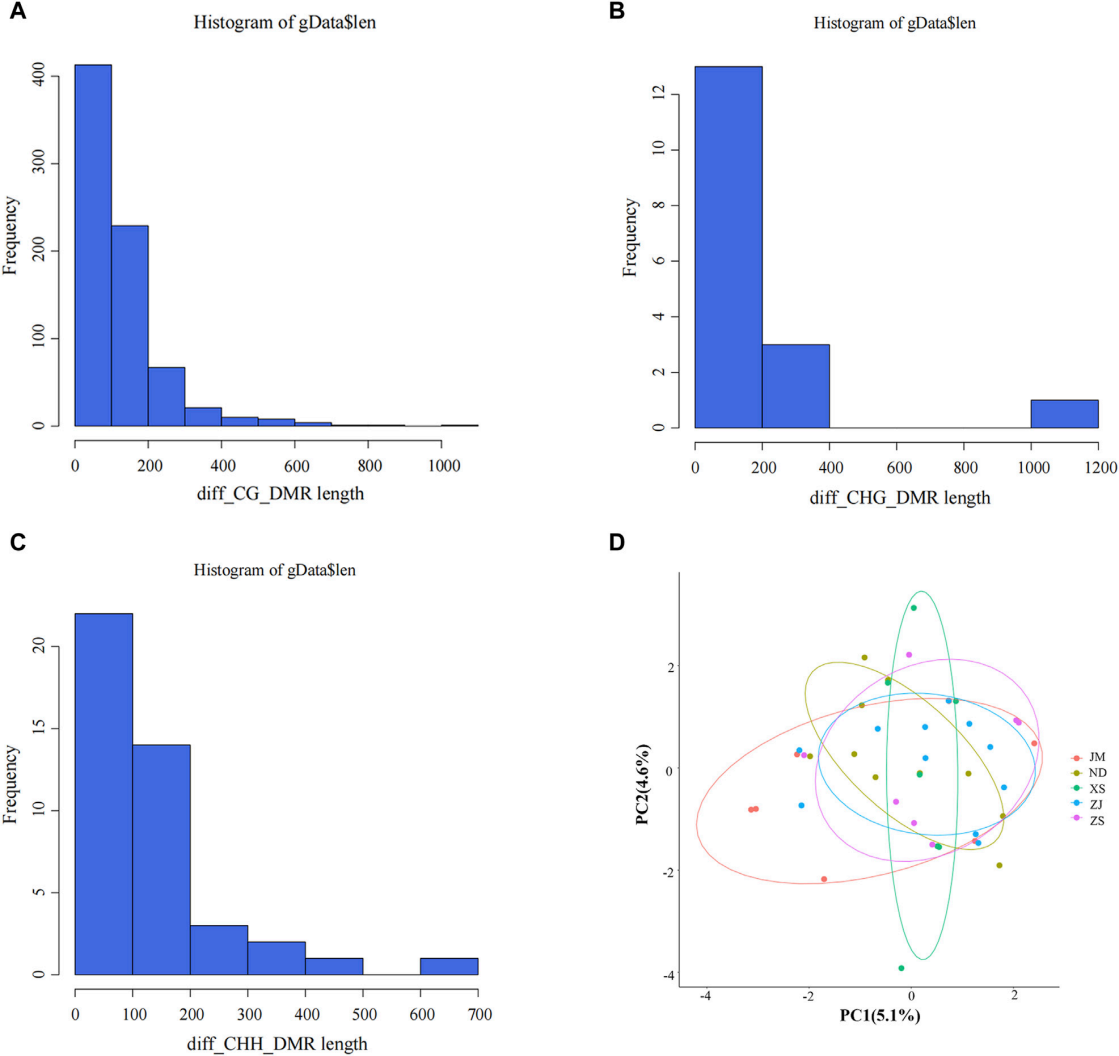
Identification of differential methylation (DMR). **(A–C)** Length distribution of DMRs. The abscissa represents the DMR length, the vertical coordinate represents the density value at each length, and black represents the distribution fitting curve.**(D)** Principal component analysis between different samples. Different colors represent different groups, ellipses represent 95% confidence intervals, and points outside the circle represent non-statistically significant.

This study identified differentially methylated regions (DMRs) between populations of *L. crocea* from different habitats. The length distribution of these regions was analyzed (Figures 2A–C), revealing that the majority of DMRs were concentrated around 200 bp, with a smaller proportion ranging from 700 to 1,200 bp. These findings indicate that the identified length range of DMRs is reasonable and provide valuable reference values for further analysis of the characteristics of differential methylation regions in different habitats.

PCA analysis was conducted on the DMRs extracted from different samples to elucidate the similarities and differences in population structure between samples and to gain insight into the degree of DMR differences among different groups (Figure 2D). The analysis revealed that the distribution of all population samples is relatively concentrated, with a few individual samples scattered, but the majority of samples fall within the confidence ellipse. This suggests that the similarity between populations is high, indirectly indicating a similar genetic background between populations and potentially similar methylation patterns. These findings hold significant background significance for subsequent DMR analysis.

### 3.4 Differential methylation analysis and annotation

**FIGURE 3:**
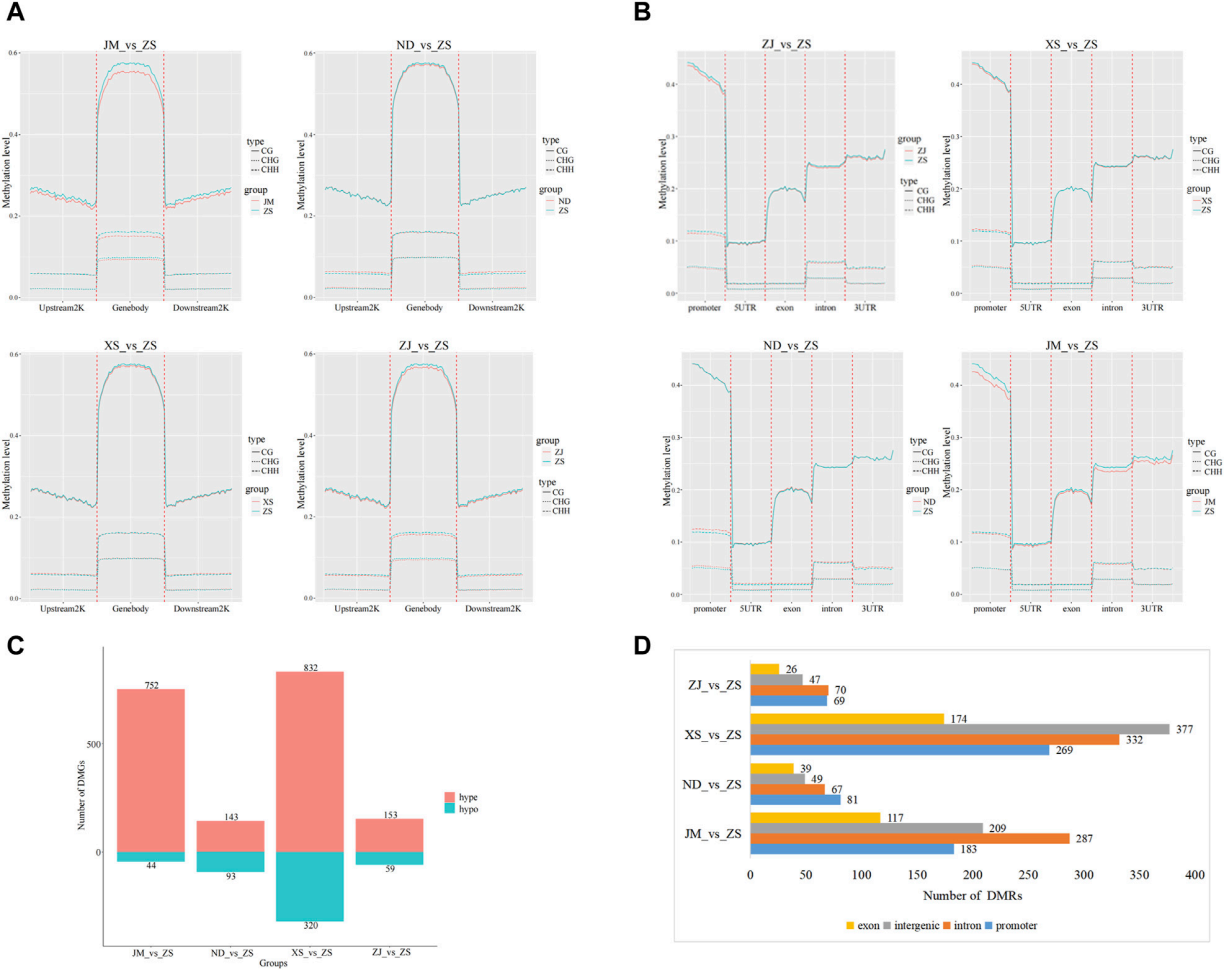
Methylation difference analysis. **(A)** Methylation levels of genes upstream and downstream among populations of largemouth yellow croaker in different habitats. **(B)** Functional area methylation levels between large yellow croaker populations in different habitats. **(C)** After comparing with ZS, the number of hypermethylated genes (hyper) and hypomethylated genes was identified by the classification statistics of differential methylation genes. **(D)** Annotation of DMRs in CG methylation mode. The overall methylation level of the intron region and the intergenic region was high, followed by the promoter region, which was consistent with the fluctuation of CG methylation levels.

The initial step involved conducting a statistical analysis of the C-site methylation levels in the gene body, upstream 2K, and downstream 2K regions of the large yellow croaker populations in different environments, followed by plotting a trend distribution graph of their methylation levels (Figure 3A). The methylation level trends in the C-site among the two comparison groups remained generally consistent across different sequence environments, with minor variations observed in the methylation levels in the gene body, upstream 2K, and downstream 2K regions of the analyzed groups. Specifically, in the CG sequence environment, significant differences in methylation levels were observed in JM compared to ND, XS, ZJ, and ZS in the gene body, upstream 2K, and downstream 2K regions, whereas the methylation levels among the other analyzed groups exhibited the opposite trend. In the CHG sequence environment, significant differences were noted in the gene body region among the other analyzed groups, such as JM_vs._ZS, while the methylation level trends in the upstream 2K or downstream 2K regions remained consistent. Conversely, in the CHH background, the methylation levels among the analyzed groups remained generally unchanged.

Furthermore, the methylation patterns between different functional regions, including promoter, 5UTR, exon, intron, and 3UTR, were compared and analyzed for all groups (Figure 3B). The results revealed consistent methylation trends among different sequence environments, while significant differences were observed in methylation levels among different functional regions. Specifically, in the comparison analysis of JM and other groups within the CG sequence context, notable variations were found in the methylation levels of functional regions, particularly in the promoter, intron, and 3UTR regions. However, in the CHH and CHG contexts, the methylation levels of different functional regions remained relatively unchanged among the groups.

In the differential methylation analysis of the skeletal muscles from various habitats of the large yellow croaker, notably between ZS and other locations, a total of 7,339,213 differentially methylated regions (DMRs) were detected. Among these, the highest proportion of DMRs was found in comparisons between ZS and the JM population, while the smallest number was observed in comparisons between ZS and the ZJ population. Subsequent analysis identified 2,396 differentially methylated genes (DMGs), which included 1,880 hypermethylated genes and 516 hypomethylated genes (Figure 3C). Notably, nearly half of the DMGs were discovered in the XS_vs._ZS comparison group. The annotation of the DMGs linked to the DMRs identified in the CG sequence environment showed that these methylation regions were predominantly located in introns (Figure 3D).

### 3.5 Analysis of differential methylated regions at gene locations

**FIGURE 4:**
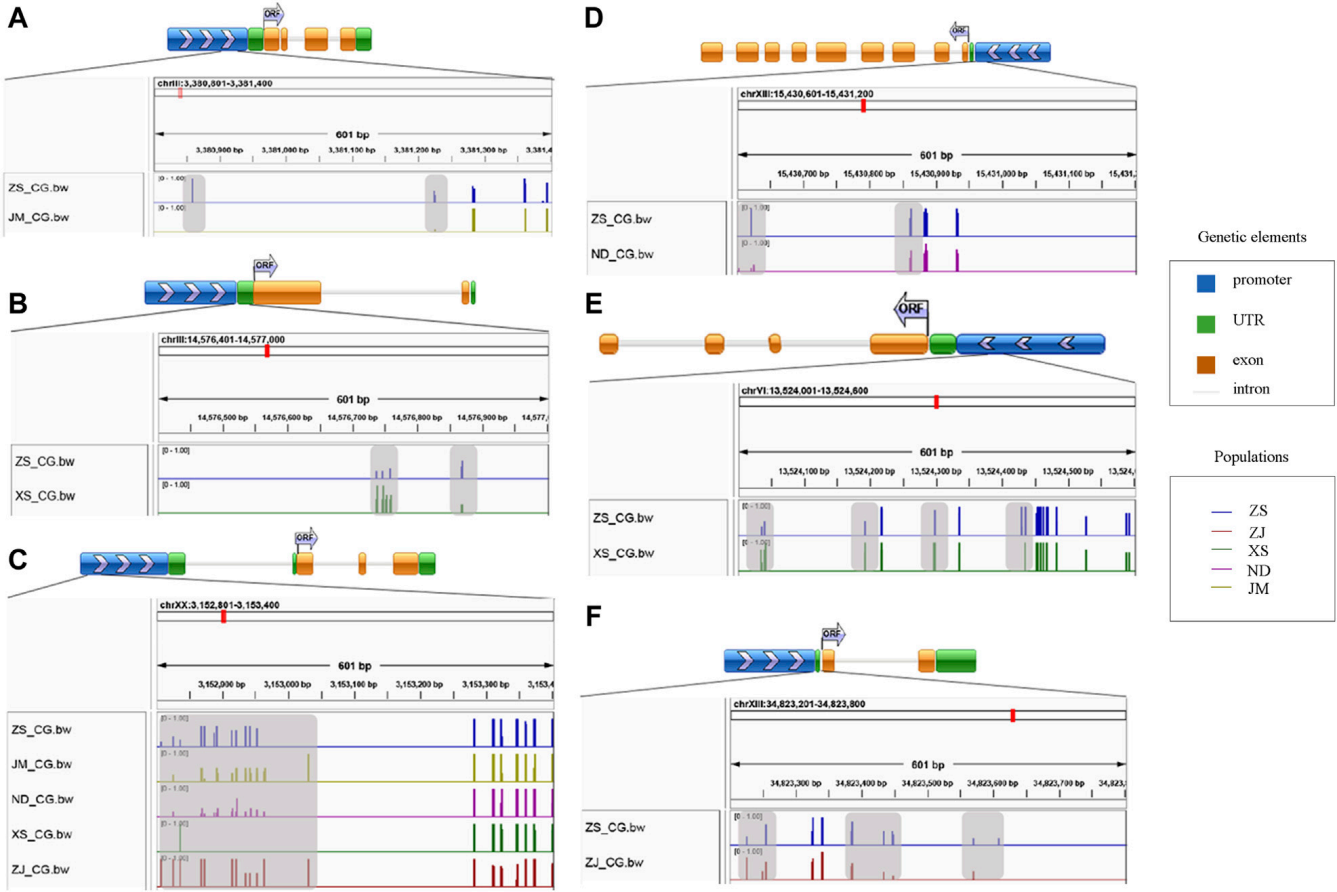
Map of differential methylation gene structure. **(A)** stand for bag4; **(B)** stands for klhl31; **(C)** stands for myf5; **(D)** stands for ndufv1; **(E)** stands for syn2b; **(F)** stands for sostdc1a; The gray area represents the region of differential methylation.

Based on the screening of differentially methylated genes related to skeletal muscle in the CG sequence environment, several genes were identified, including *myh10*, *myf5*, *myf6*, *ndufv1*, *klhl31*, *map3k4*, *syn2b*, *sostdc1a*, *bag4*, and *hsp90ab*. Gene structure diagrams were generated based on predictions from online software (Figure 4). The differentially methylated sites in these genes were predominantly located in the promoter and exon regions, with varying methylation levels observed among different populations. For instance, significant differences in methylation levels were noted in the promoter region of the *myf5* gene across different populations, particularly in XS, where the methylation level was low or unmethylated. In the *klhl31* gene, differential methylation occurred in both the promoter and the first exon regions, with notable differences between ZS and XS, where ZS exhibited a lower methylation level. In *bag4* and *ndufv1*, differential methylation was observed in regions adjacent to the exon, with ZS displaying a higher methylation level compared to other populations. Additionally, the differential methylation regions in *syn2b* and *sostdc1a* also occurred in the promoter region, albeit with varying methylation levels at different positions. High methylation in the promoter region may inhibit gene expression regulation, while methylation in the exon region could activate gene expression.

### 3.6 Analysis of differential methylation genes GO and KEGG

**FIGURE 5:**
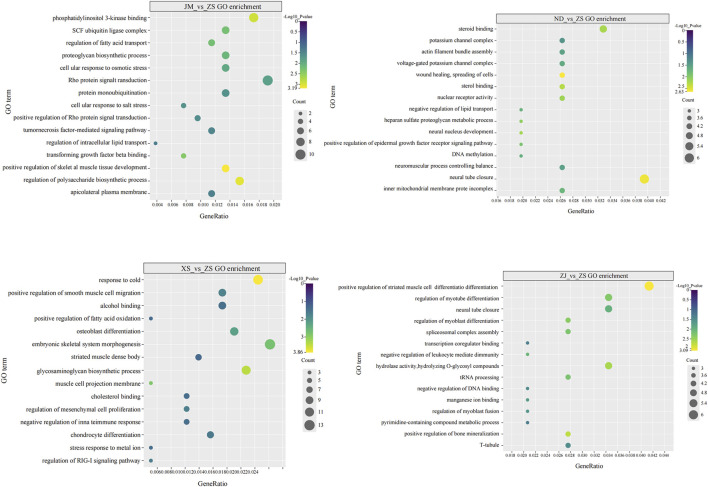
GO enrichment plot of DMGs, which mainly related to the growth and development of skeletal muscle.

This study conducted GO functional classification and KEGG pathway annotation and enrichment analysis on DMGs, identifying 626 significantly enriched GO functional categories and 10 KEGG pathways. Multiple muscle-related genes were found to be enriched in the GO and KEGG enrichment analysis of DMGs. The enriched GO functional categories in the comparison between ZS and other populations were primarily related to muscle system development (Figure 5). Moreover, the enriched GO functional categories in the five populations encompassed developmental and environmental stress-related processes, including regulation of cell response to osmotic stress (GO:0071470), positive regulation of skeletal muscle tissue development (GO:0048643), DNA methylation (GO:0006306), and cold acclimation (GO:0009409), among others. These findings provide valuable insights into the molecular mechanisms underlying muscle development and environmental adaptation in *L. crocea* populations.

The KEGG enrichment results of ZS compared to other populations (Table 2) revealed significant enrichment of genes in pathways related to biosynthesis, biological rhythm, and immune signal transduction. Notably, the analysis group of JM_vs._ZS had the most pathways enriched with differentially methylated genes, while ZJ_vs._ZS had the least. Among the enriched pathways were the TGF-β signaling pathway (ko04350) related to fibroblast transformation, chondroitin sulfate synthesis (ko00510), immune (ko04624), and repair mechanism (ko03450), among others. These findings indicate that the pathways regulated during the growth and development of skeletal muscles in different populations may vary due to differences in the differentially methylated genes, which could be attributed to their respective living environments.

**TABLE 2:** KEGG enrichment statistics of skeletal muscle in each analysis groups.

Groups	Id	Description	*P* value	Count
JM_vs._ZS	ko05217	Basal cell carcinoma	0.000626821	9
	ko00512	Mucin type O-glycan biosynthesis	0.003902303	5
	ko03450	Non-homologous end-joining	0.012337284	3
	ko00532	Glycosaminoglycan biosynthesis–chondroitin sulfate/dermatan sulfate	0.021925117	3
	ko04624	Toll and imd signaling pathway	0.022356081	7
	ko04350	TGF-beta signaling pathway	0.045413519	8
ND_vs._ZS	ko04710	Circadian rhythm	0.019549152	3
	ko00534	Glycosaminoglycan biosynthesis–heparan sulfate/heparin	0.025832987	2
	ko05217	Basal cell carcinoma	0.030527944	3
	ko03020	RNA polymerase	0.048477982	2
XS_vs._ZS	ko04710	Circadian rhythm	0.019549152	3
	ko00534	Glycosaminoglycan biosynthesis–heparan sulfate/heparin	0.025832987	2
	ko05217	Basal cell carcinoma	0.030527944	3
	ko03020	RNA polymerase	0.048477982	2
	ko04710	Circadian rhythm	0.019549152	3
	ko00534	Glycosaminoglycan biosynthesis–heparan sulfate/heparin	0.025832987	2
	ko05217	Basal cell carcinoma	0.030527944	3
ZJ_vs._ZS	ko03450	Non-homologous end-joining	0.009160256	2
	ko00510	N-Glycan biosynthesis	0.021220134	3
	ko05144	Malaria	0.04328677	3

## 4 Discussion

The research landscape surrounding the genetic, environmental, and physiological factors influencing miRNA and DNA methylation expression in fish muscles has expanded significantly in recent years. Studies have demonstrated that these factors contribute to differences in growth response and muscle phenotypic variation in fish, enabling them to adapt swiftly to environmental changes. Notably, investigations in fish model organisms such as *D. rerio* (Lin et al., 2013), *Gasterosteus aculeatus* (Craig and Moon, 2013), *Oncorhynchus mykiss* (Canada et al., 2019), *Salmo salar* (Burgerhout et al., 2017), *Dicentrarchus labrax* (Anastasiadi et al., 2017), and *Oreochromis niloticus* (Podgorniak et al., 2019) have provided valuable insights into this phenomenon. However, research on non-model fish species, particularly regarding DNA methylation in the muscles of different populations of *L. crocea* across various habitats, remains limited. This paper aims to bridge this gap by analyzing the differences in methylation patterns among *L. crocea* populations inhabiting different habitats. By doing so, the study seeks to enhance our understanding of the selective adaptation of organisms to diverse habitats and the variations in environmental adaptation resulting from domestication selection.

The construction of a whole-genome DNA methylation map of *L. crocea* muscle tissue revealed significant differences among the three types of methylation: CG, CHG, and CHH. While CHG and CHH methylation sites were more widely distributed across the genome, their overall methylation levels were lower, suggesting a potential role in maintaining genome stability (Nardo et al., 2015). Conversely, CG methylation sites, although fewer in number, exhibited higher methylation levels, indicating their importance in epigenetic regulation of gene expression (Suzuki and Bird, 2008; Jones, 2012). These CG methylation sites likely contribute to processes such as muscle growth and development, myocyte differentiation, muscle contraction, and myotube cell differentiation (Chu et al., 2017; Mishima et al., 2009; O’Rourke et al., 2007). Identification of differentially methylated regions (DMRs) revealed substantial methylation differences between various populations, with the largest disparities observed between JM_vs._ZS and XS_vs._ZS. These findings suggest that *L. crocea* populations have undergone natural selection and genetic variation in response to different environmental conditions. Moreover, during the transition from natural to artificial feeding, the species experienced intense reproductive environments and domestication selection, leading to significant epigenetic changes in DNA methylation to facilitate adaptation. Interestingly, while no significant differences were found among *L. crocea* populations in CG, CHH, and CHG sequence environments, DMRs of CG sequence type exhibited notable differences in methylation levels across gene elements. This underscores the importance of whole-genome DNA methylation in influencing gene expression regulation in various genomic regions, such as promoter regions for gene expression regulation and exon regions for gene expression activation (Kass et al., 1997; Smith and Meissner, 2013).

Enrichment analysis revealed that differentially methylated regions (DMRs) in *L. crocea* were associated with various pathways linked to muscle development, including “positive regulation of striated muscle cell differentiation”, “response to cold”, “positive regulation of fatty acid oxidation”, “regulation of RIG-I signaling pathway”, “Rho protein signal transduction”, and “positive regulation of skeletal muscle tissue development”. These findings suggest a potential role for CG sequence DNA methylation in shaping the muscle system in *L. crocea* populations. Among the identified differentially methylated genes implicated in muscle growth and development are *myf5/6*, *ndufv1*, *klhl31*, *sostdc1a*, and *myh10*. Notably, *myf5/6*, members of the MRF gene family, serve as transcriptional regulatory factors (Yang et al., 2012). Their differential methylation patterns may influence the expression of muscle-specific genes, contributing to the intricate regulatory network underlying muscle differentiation and development. In this study, myf5/6 was identified as a protein-coding gene, and its binding to DNA may be associated with hypo-DMRs in the promoter region, potentially regulating the expression of muscle-specific genes. Recent research has highlighted the role of specific structural or regulatory gene DNA methylation and demethylation in orchestrating skeletal muscle development (Simó-Mirabet et al., 2020). Notably, studies on *S. salar* have revealed a correlation between high myogenin expression and relatively low DNA methylation levels (Burgerhout et al., 2017). Another gene of interest, ndufv1 (NADH: Ubiquinone Oxidoreductase Core Subunit V1), serves as a core subunit of mitochondrial complex I. In this investigation, DNA methylation was observed in the promoter region of the ndufv1 gene, potentially leading to the inhibition of ndufv1 production. Interestingly, studies involving mouse models have demonstrated that knockout of the ndufv1 gene in muscle tissues results in increased muscle generation (Garvin et al., 2015). These findings underscore the intricate regulatory role of DNA methylation in modulating gene expression and physiological processes associated with muscle development and function.

In *O. mykiss*, the transcription of ndufv1 was observed to be upregulated under temperature stress, suggesting that this gene may play a role in the organism’s response to environmental changes (Hong et al., 2014). Additionally, klhl31, a member of the Kelch-like protein family in vertebrates, is implicated in regulating myogenesis by interacting with myogenic signals such as Shh and Wnt-1 or Wnt-6 signaling factors (Abou-Elhamd et al., 2009). Furthermore, the myh10 gene encodes a member of the myosin superfamily, which functions as a conventional non-muscle myosin. While muscle myosin is a movement protein dependent on actin, myh10 serves various roles including regulating cytoplasmic division, cell movement, and cell polarity (Zhang et al., 2014). These genes likely contribute to the physiological adaptation of large yellow croaker to diverse environmental conditions.

In addition, the sostdc1a gene is a member of the sclerostin protein family, encoding a N-glycosylated secreted protein with a C-terminal cysteine knot domain. Studies have found that *sostdc1a* is an upregulated gene in *O. mykiss* under cold stress, and is considered a general biomarker for cold shock (Borchel et al., 2017). Bag4 is a protein-coding gene that plays a role by interacting with various cell death and growth-related proteins, and regulates their activity by binding to *hsp70/hsc70* family proteins (Brünger et al., 1998). syn2b is a member of the synaptotagmin gene family, encoding a neuronal phosphoprotein associated with synaptic vesicle cytoplasmic surfaces (See et al., 2014). *map3k4 is* also a protein-coding gene, and studies have shown that high methylation of *map3k4* kinase and low methylation of uchl3, observed in seawater-released marine sticklebacks, can potentially reduce protein ubiquitination levels and serve as an adaptation to osmotic stress (Artemov et al., 2017). Therefore, the differential methylation patterns of these genes suggest that epigenetic modifications may affect their subsequent expression, and thus participate in and promote the adaptation of the *L. crocea* to artificial domestication and environmental selection in different habitats.

The study conducted whole-genome DNA methylation sequencing on farmed and wild-caught *L. crocea* populations, yielding 622.07 Gb of raw data. Through differential methylation analysis, it was observed that while the methylation patterns in functional regions and upstream/downstream regions of genes were largely consistent across different populations, significant variations existed in methylation levels. A total of 2,396 differentially methylated genes (DMGs) were identified among *L. crocea* populations from five distinct habitats. Enrichment analysis revealed that these DMGs were predominantly associated with muscle growth and development, encompassing crucial developmental genes linked to phenotype alterations. This research offers valuable genetic insights for marine fish aquaculture by elucidating the influence of epigenetic modifications on artificial selection and environmental adaptation.

## Data Availability

The data presented in the study are deposited in the NCBI (https://www.ncbi.nlm.nih.gov/) repository, accession number PRJNA1124228.
